# Gradient Guided-Mode Resonance Biosensor with Smartphone Readout

**DOI:** 10.3390/bios13121006

**Published:** 2023-11-29

**Authors:** Ting-Zhou Lin, Cheng-Hao Chen, Yuan-Pei Lei, Cheng-Sheng Huang

**Affiliations:** Department of Mechanical Engineering, National Yang Ming Chiao Tung University, Hsinchu 30010, Taiwan; a309611023.en09@nycu.edu.tw (T.-Z.L.); ohyes12321.me07g@nctu.edu.tw (C.-H.C.); bernardlei.en11@nycu.edu.tw (Y.-P.L.)

**Keywords:** smartphone biosensor, optical biosensor, guided-mode resonance, albumin, creatinine

## Abstract

Integrating biosensors with smartphones is becoming an increasingly popular method for detecting various biomolecules and could replace expensive laboratory-based instruments. In this work, we demonstrate a novel smartphone-based biosensor system with a gradient grating period guided-mode resonance (GGP-GMR) sensor. The sensor comprises numerous gratings which each correspond to and block the light of a specific resonant wavelength. This results in a dark band, which is observed using a CCD underneath the GGP-GMR sensor. By monitoring the shift in the dark band, the concentration of a molecule in a sample can be determined. The sensor is illuminated by a light-emitting diode, and the light transmitted through the GGP-GMR sensor is directly captured by a smartphone, which then displays the results. Experiments were performed to validate the proposed smartphone biosensor and a limit of detection (LOD) of 1.50 × 10^−3^ RIU was achieved for sucrose solutions. Additionally, multiplexed detection was demonstrated for albumin and creatinine solutions at concentrations of 0–500 and 0–1 mg/mL, respectively; the corresponding LODs were 1.18 and 20.56 μg/mL.

## 1. Introduction

Since 2004, smartphones have become indispensable devices for people worldwide [1]. The data storage, wireless communication, high-resolution imaging, and high-speed computing capabilities of smartphones are continually increasing, and these devices have numerous sensors, such as vibration, rotation, and acceleration sensors, cameras, and satellite navigation receivers. Some researchers have integrated custom hardware and software into smartphones for various applications, including medical tests. The current smartphone biosensor systems can be classified into four categories in accordance with their measurement or detection target, namely, cell or pathogen detection, biochemical reaction detection (e.g., blood sugar detection), immune detection, and molecular detection (e.g., DNA) [2]. They can also be classified by their detection mechanism into microscope (e.g., bright field) [3,4] fluorescence [5,6], electrochemical [1,7,8], reagent test paper [9,10,11] or colorimetric [12,13,14], and lateral flow assay [7,15] detection systems. Smartphone spectrometers have also been developed that act as signal readout devices for several optical biosensors [16,17,18,19,20].

For each detection mechanism, different detection instruments are required to measure the target physical quantities. Recently, researchers have developed various smartphone-integrated readout or measurement devices. The majority of studies on smartphone-integrated biomolecule detection methods have focused on fluorescence and colorimetrical detection [5,6,7,9,10,11,12,13,14,15]. In these applications, the smartphone is used for image capture and subsequent analysis to quantify the variation in the color of a test strip or sample. Researchers have mainly focused on designing a fixture or cradle for holding the smartphone, other necessary components, or user interface software [9,10,11,12,13,14,15]. Electrochemical biosensors usually use surface-modified electrodes that react with specific molecules (e.g., blood sugar, uric acid, and blood ketones). To integrate these biosensors with smartphones, an external circuit module is often required to read out the sensor signal, which varies in accordance with the concentration of the detected molecules. Three detection mechanisms are commonly used: those based on potential, impedance, and current [21,22]. Conventional electrochemical biosensors have shortcomings, including poor stability and reproducibility [23]. In contrast, optical biosensors have various advantageous capabilities, namely, high sensitivity, remote detection, real-time monitoring, multiplexing, a simplified design, and immunity from electromagnetic interference. In contrast to that of electrochemical biosensors, the performance of optical biosensors is less strongly affected by the pH of reagents. Hence, optical biosensors have been widely used in the detection of various biomolecules.

Surface plasmon resonance (SPR) and localized SPR (LSPR) sensors are the optical biosensors most commonly integrated with smartphones. Preechaburana et al. [24] designed a polydimethylsiloxane (PDMS) optical coupling mechanism in which various optical components are integrated with an SPR chip. The PDMS coupling mechanism allows the light from the smartphone screen to be coupled into the SPR chip and subsequently couples the reflected light from the SPR chip into the smartphone’s camera. The light intensity was then measured with a CCD. The detection mechanism was based on angle-resolved intensity modulation; this method requires a complex optical path design and has demanding requirements regarding the alignment between the coupling mechanism and the smartphone.

Bremer et al. [17] and Liu et al. [25] avoided the need for a free-space optical design by using optical fibers for SPR detection; in both studies, the smartphone’s built-in flash was employed as the light source. In 2018, Zhang et al. [16] used a grating-coupled SPR chip with a custom-made optomechanical coupling device that enabled the flash light to be coupled with the SPR chip. The reflected light was transmitted through a diffraction grating which converted the spectral information into pixel information that could be captured using the CCD and allowed the resonant wavelength to be calculated. Walter et al. [18] used an optical waveguide SPR chip instead of optical fibers, creating a smaller optomechanical device.

Roche et al. [26] and Dutta et al. [20] employed the characteristics of LSPR of metal nanoparticles to detect biomolecules in suspension assays. The detection mechanism was based on the resonant wavelength shift upon the metal nanoparticles’ immobilized capture probes binding with the analyte. Fan et al. [19] developed an LSPR-based array of nine sensors containing randomly distributed gold nanoparticles. In addition, they produced a custom smartphone attachment with an embedded grating that incorporated the sensor array and other components.

Researchers have also investigated detection modalities other than the common intensity- or wavelength-based methods. Cetin et al. [27] developed an SPR-based smartphone biosensing system in which a light-emitting diode (LED) light source was used to illuminate the sensor in a nanoarray structure. The variation in the diffraction image formed in the CCD was then used to quantify the concentration in a sample. The achieved limit of detection (LOD) was lower than those achieved with other techniques; however, the system uniquely did not require any additional optics—the algorithm alone enabled the calculation of the variation in the diffraction image and the corresponding intensity.

The first smartphone biosensor based on photonic crystals was developed by Gallegos et al. [28]. Detection was achieved through spectral measurements realized using a diffraction grating, camera lens, CCD, and external white light. The shift of the resonant wavelength of the photonic crystal upon sample loading was monitored to determine the concentration of the target substance. Giavazzi et al. [29] designed a custom smartphone cradle and used its flash as a light source to illuminate a reflective phantom interface (RPI). The reflected light was captured by the smartphone’s camera, and the intensity was recorded by a CCD. The reflected intensity was affected when an analyte was bound to the RPI; this intensity was correlated with the analyte concentration. Although the detection mechanism was simple, the setup was somewhat complex.

Table 1 presents a summary of the aforementioned optical label-free biosensor smartphone systems, including their optomechanical and attachment designs, sensor type, detection modality, assays and detection limit, and other characteristics. Many designs leverage either the wavelength or intensity modulation for detection and require an external grating or prism for spectral analysis.

In this work, we demonstrate a new type of smartphone biosensor system based on a gradient grating period (GGP) guided-mode resonance (GMR) biosensor [30,31]. The system works as follows: (1) An external LED is used to excite the resonance of the GGP-GMR sensor at normal incidence, simplifying the overall optical path design and minimizing the attachment volume. (2) The GGP-GMR sensor directly converts the spectral information into spatial information on the CCD; a dispersive element such as a prism or diffraction grating is not required, facilitating smartphone integration. (3) The measurement is based on the relative shift in the intensity distribution instead of a single intensity value; therefore, the measurement is self-referencing and unlikely to be affected by environmental fluctuations, ensuring high accuracy. The GGP-GMR was first proposed as a linear variable bandstop filter [30] and then demonstrated as a refractive index sensor [31] and biosensor [32]. In this study, we developed a new design and fabrication process to make a 2 × 2 GGP-GMR sensor array. For the first time, multiplexing detection with a GGP-GMR sensor array was demonstrated and directly read out by a smartphone.

**Table 1:** Summary of optical biosensor smartphone systems.

Mech	Sensor	Det Mod	Light Source	Assay	DR	LODA_A_	Remarks	R
SPR	Au film	ARI	Phone screen	β_2_M	0.132–1.32 μg/mL	0.1 μg/mL	PDMS coupling device	[24]
SPR	Au nanohole array	PRI	Flash	IgG	3.9–1000 μg/mL	μg/mL	Lens free	[27]
SPR	Ag-coated fiber	λ	Flash	–	–	–	Grating	[17]
SPR	Au-coated fiber	I	Flash/NB	SPA	67–1000 nM	47.4 nM	–	[25]
SPR	Au-coated grating film	λ	Flash	LPS	0–10 μg/mL	32.5 ng/mL	Grating	[16]
SPR	Au-coated waveguide	λ	LED	Vit D	0–100 nM	25 nM	Grating	[18]
SPRi	Ag/Au-coated grating bilayer film	I	LED	IgG	1.3–830 nM	~nM	Array	[33]
LSPR	Suspension AuNPs		LED	CCL2	0.099–1 μg/mL	0.099 μg/mL	Grating and cuvette	[26]
LSPR	Suspension AuNPs	λ	BB	–	–	–	Grating and cuvette	[20]
LSPR	Random AuNP film	λ	LED	CA125 CA153	–	4.2 0.87 U/ml	Grating	[19]
PC	1-D PC	λ	BB	IgG	4.25–3400 nM	4.25 nM	Grating	[28]
RPI	RPI surface	I	LED	p24		10 ng/ml	Prism	[29]

Mech, mechanism; Det Mod, detection modality; SPR, surface plasmon resonance; SPRi, surface plasma resonance imaging; LSPR, localized surface plasma resonance; PC, photonic crystal; RPI, reflective phantom interface; ARI, angle-resolved intensity; PRI, phase-resolved intensity; R, reference; I, intensity λ, wavelength; NB, narrowband filter; BB, broadband; Vit D, vitamin D; LOD_B_, limit of detection from bulk solution; LOD_A_, limit of detection from assay; DR, detection range; and AuNPs, gold nanoparticles.

## 2. Materials and Methods

### 2.1. Design and Fabrication of GGP-GMR Sensor Array

A GMR filter of appropriately selected dimensions and materials functions as a bandstop filter [34,35,36]. At normal incidence, light of a specific wavelength resonates with a structure (of resonance wavelength λ) and is reflected; light of other wavelengths is transmitted. The reflected (or resonant) wavelength can be calculated based on the second-order Bragg condition [37]:

λ = n_eff_Λ (1)

where n_eff_ is the effective refractive index (RI) of the structure and Λ is the grating period. n_eff_ can be considered as a weighted RI, which is related to the thickness of the waveguide layer and the RIs of the substrate, waveguide, and cover (or sample solution) layer.

**Figure 1:**
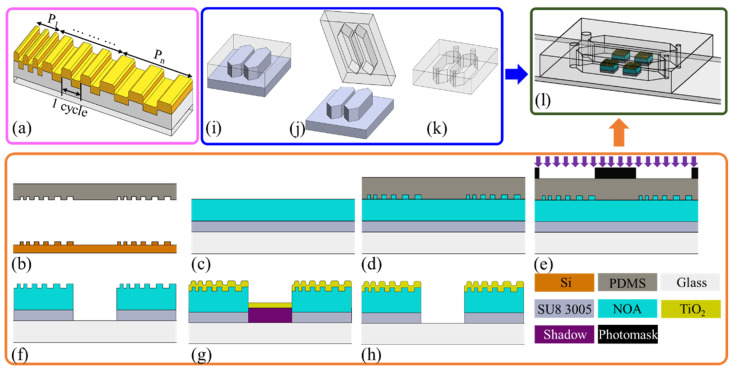
(**a**) GGP-GMR periods. Processes for fabricating the (**b**–**h**) GGP-GMR sensor array and (**i**–**k**) microfluidic channel. (**l**) Schematic of the integrated sensor chip.

Conventional GMR filters have a constant grating period. By contrast, the GGP-GMR system proposed in this work has grating periods of between 370 and 390 nm in 2 nm increments and each period is repeated 100 times (Figure 1a; only three times are shown for simplicity). In this work, each period was repeated for 100 cycles. We tried 50, 150, and 200 cycles previously. Our observations indicated that with only 50 cycles, the resonant efficiency is diminished, resulting in a less distinct dark band. This could pose a challenge for subsequent image analysis and potentially compromise detection resolution. Conversely, employing 150 or 200 cycles results in a sensor that is too large, possibly exceeding the field of view. When light of a specific wavelength illuminates the GGP-GMR sensor, the light resonates at a specific period in accordance with Equation (1); hence, the light is reflected back from this (resonant) period but transmitted through other periods. In the CCD underneath the GGP-GMR sensor, the pixel underneath the resonant period is exposed to the lowest intensity of light. If the concentration in the sample on top of the GGP-GMR sensor changes, the RI and, therefore, n_eff_ changes; hence, the resonant period for the same incident wavelength changes in accordance with Equation (1). The minimum-intensity pixel shifts accordingly and the magnitude of this shift is correlated with the change in the sample’s RI.

An integrated sensor chip comprising a 2 × 2 GGP-GMR sensor array embedded in two microfluidic channels was fabricated to demonstrate the proposed biosensor’s potential multiplexing capability and smartphone integration. The overall fabrication process comprised the fabrication of the sensor array (Figure 1b–h), fabrication of the microfluidic channels (Figure 1i–k), and bonding (Figure 1l).

The GGP-GMR sensor array was fabricated through four main processes, namely, electron beam (e-beam) lithography, nanoimprinting, photolithography, and film deposition (Figure 1b–h). In brief, e-beam lithography and dry etching were used to generate a gradient grating pattern on a Si wafer; subsequently, the pattern was replicated on a PDMS master (Sylgard 184, Dow Corning, MI, USA; Figure 1b). The Si mold was treated with repel silane (PlusOne Repel-Silane ES, GE Healthcare) to prevent it from sticking to the PDMS. After surface silanization was performed, the PDMS was used to replicate the GGP patterns. A base resin and curing agent in a 7:1 ratio were thoroughly mixed and applied to the Si mold, which was placed in a vacuum desiccator to remove air bubbles. Once the liquid PDMS had solidified in an oven at 100 °C for 1 h, the cured PDMS was separated from the Si mold, as illustrated in Figure 1b.

The PDMS master was then used to transfer the grating pattern to produce the final sensor array. A glass slide was cleaned through sonication in solutions of acetone, isopropanol (IPA), and deionized (DI) water for 10 min each. A layer of SU8 3005 (negative photoresist) was then spin-coated onto the glass slide at 2000 rpm for 30 s; the slide was subsequently soft-baked at 90 °C for 2 min on a hotplate. An optical adhesive (Norland 68, NOA) was applied to the top of the SU8 3005, as depicted in Figure 1c. The PDMS master was gently pressed against the NOA (Figure 1d) to imprint the grating pattern. The SU8/NOA was exposed using an i-line contact aligner (Kyowa Riken) at 24,000 mJ/cm^2^ through a photomask with a 2 × 2 opening (2 × 2 mm^2^), as shown in Figure 1e. After this exposure, the PDMS was separated from the NOA, and the whole glass slide was developed in propylene glycol methyl ether acetate (PGMEA) to dissolve the unexposed region (Figure 1f). A layer of TiO2 (~130 nm) was sputtered on the imprinted NOA region by using a shadow mask (Figure 1g). The shadow mask was then removed, completing the 2 × 2 GGP-GMR sensor array (Figure 1h).

**Figure 2:**
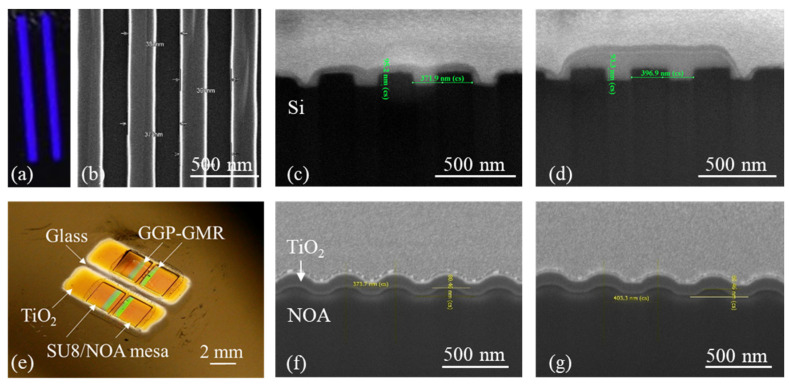
(**a**) Picture of the Si mold with two gradient grating patterns. (**b**) Top-view SEM image of the Si mold at the smallest period. SEM cross-sectional views of the Si mold at the smallest (**c**) and largest period (**d**). (**e**) Photograph of the 2 × 2 GGP-GMR sensor array on top of the NOA/SU8 mesa. SEM cross-sectional views of the final grating on the GGP-GMR sensor at the smallest (**f**) and largest period (**g**).

The grating pattern on the GGP-GMR sensor was produced by performing two pattern transfers: PDMS replication from an original Si mold and PDMS imprinting on NOA. Figure 2a depicts an image of the Si mold fabricated through e-beam lithography, featuring two gradient grating patterns, each with a length of 1.2 cm. Figure 2b presents a top-view scanning electron microscopy (SEM) image of the Si mold at the smallest period. Figure 2c,d present SEM cross-sectional images of the original Si mold (illustrated in Figure 1b). Figure 2e presents a photograph of the fabricated 2 × 2 GGP-GMR sensor on the SU8/NOA mesa. The shadow mask used for sputter deposition enabled the deposition of TiO_2_ on the grating/mesa region, resulting in a gap and a glass surface surrounding the mesa. This surface was required for the subsequent bonding with PDMS microfluidic channels through an oxygen plasma treatment technique. The SEM cross-sectional images of the final TiO_2_/NOA grating on the mesa are shown in Figure 2f,g, illustrated in Figure 1h. The resulting grating pattern deviates from that of the original Si mold in several aspects, namely, its surface profile (sinusoidal vs. rectangular), shallower grating depth, and rounded corners. The Si mold exhibits periods ranging from 371.2 to 396.9 nm with a grating depth of 93.3 nm, as shown in Figure 2c,d. By contrast, the resulting periods for the GGP-GMR sensor on the NOA substrate range from 373.7 to 403.3 nm with an approximate grating depth of 81.6 nm, as depicted in Figure 2f,g.

**Figure 3:**
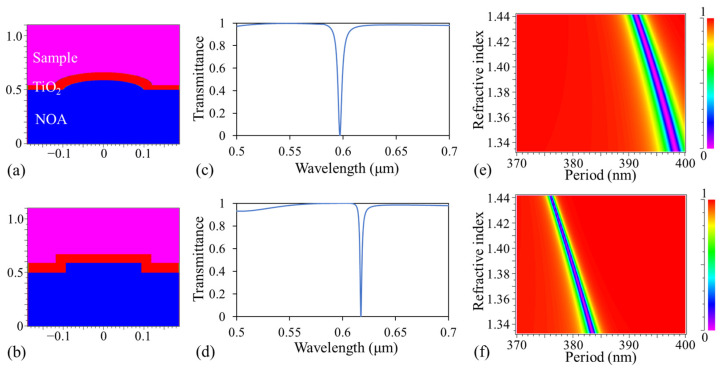
Cross-section of one period of GMR with rounded corners (**a**) and with rectangular corners (**b**) used in the DiffractMOD simulation. The transmission spectrums of the rounded corners (**c**) and rectangular corners (**d**) for a grating period and depth of 370 nm and 90 nm, respectively. The transmission efficiency of a specific sample RI at a specific period with an incident wavelength of 630.5 nm for the rounded corners (**e**) and rectangular corners (**f**).

To assess the impact on the deviation of the grating profile, we employed the simulation tool DiffractMOD from RSoft Design Group, which is based on rigorous coupled-wave analysis, to estimate the sensor performance. Based on the SEM images from Figure 2, two types of models were constructed in the simulation: one with rounded corners (Figure 3a) and the other with rectangular corners (Figure 3b). First, both models used a grating period of 370 nm, a grating depth of 90 nm, and a TiO_2_ thickness of 90 nm for comparing the resonance bandwidth as a typical GMR device. RIs of 1.33299, 2.29, and 1.556 were assigned to the sample, TiO_2_, and NOA, respectively. The transmission spectra are presented in Figure 3c,d for rounded and rectangular corners, respectively, with resulting full width at half maximum (FWHM) values of 4.41 and 2.03 nm, respectively. Secondly, by fixing the wavelength at 630.5 nm and scanning both the period from 370 to 400 nm and the RI of the sample from 1.33299 (0% sucrose solution) to 1.4418 (60% sucrose solution), we obtained the transmission efficiency as a function of the period at different sample RIs. This is illustrated in Figure 3e,f for rounded and rectangular grating, respectively. The detailed steps for constructing Figure 3e,f can be found in a previous publication [31]. The color in Figure 3e,f signifies the transmission efficiency; thus, for each sample (or RI), there exists a corresponding period (resonant period) associated with the minimum transmission efficiency. Hence, the resulting dark band can be observed with the CCD or CMOS underneath the GGP-GMR. Referring to Figure 3c–f, it can be concluded that the GMR with rectangular corners yields a narrower resonance, and the GGP-GMR with rectangular corners results in a narrower dark bandwidth. This holds the potential to enhance detection resolution and improve the LOD. Despite these deviations, the resonance (dark band) was still detectable in the smartphone image. Nevertheless, these deviations led to a slight broadening of the dark bandwidth and a reduction in detection resolution.

### 2.2. Design and Fabrication of Microfluidic Channel

**Figure 4:**
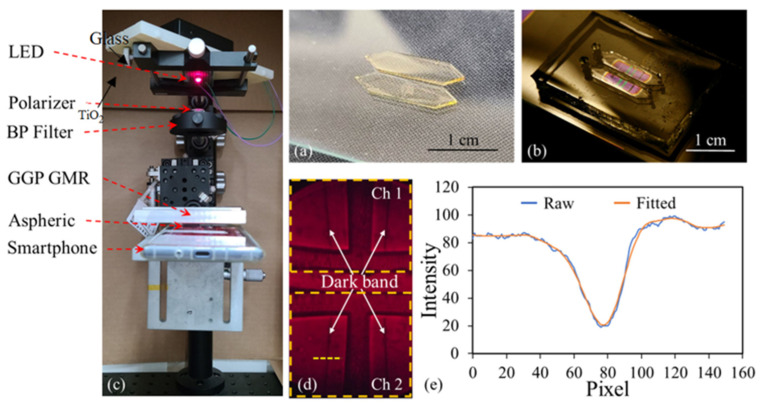
(**a**) SU8 microfluidic mold, (**b**) integrated sensor chip, (**c**) experimental setup, and (**d**) an image taken by a smartphone. (**e**) Intensity distribution along the yellow dashed line in (**d**) and the fitted curve.

A two-channel microfluidic chip was fabricated using photolithography and PDMS replica molding for sample delivery and measurement to demonstrate multiplexing detection. First, photolithography was used to fabricate a complementary SU8 mold on a glass slide. In brief, a glass slide was cleaned, as described previously, and subsequently spin-coated with SU8 2100 at 1500 rpm for 30 s. The SU8 2100/glass slide was then placed on a leveled table for 24 h for further planarization. The SU8 2100 was next soft-baked on a hotplate at 65 °C for 10 min and then at 95 °C for 100 min. These processes were repeated two more times before the sample was exposed to the same contact aligner at a dosage of 900 mJ/cm^2^. After this exposure, the SU8 2100/glass slide was post-baked on a hotplate at 65 °C for 5 min and then at 95 °C for 30 min before it was developed in PGMEA for 30 min. To further enhance the mechanical strength of the SU8, the SU8 2100/glass slide was hard-baked at 150 °C for 10 min to complete the complementary SU8 mold, which is shown in Figure 4a.

The liquid PDMS mixture was then poured into the SU8 mold (Figure 1i). After curing, the PDMS microfluidic channel was separated from the SU8 mold (Figure 1j). Lastly, the inlet and outlet holes were punched using a biopsy punch for sample injection and aspiration (Figure 1k). To bond the PDMS microfluidic channel and GGP-GMR sensor array, both chips were treated with oxygen plasma (PDC-32G, Harrick Plasma) to generate silanol groups on the PDMS and glass surfaces. The exposed surfaces were then brought into contact to create an irreversible bond through permanent Si–O–Si bonds [38] (Figure 1l). Figure 4b presents a photograph of the integrated chip with GGP-GMR sensors embedded in the microfluidic channels.

### 2.3. Assay Protocol

Immunoassay was used for biomolecule detection. Therefore, the surface of the GGP-GMR sensor array was first modified for antibody immobilization before it was bonded with the microfluidic channels as described previously. Epoxy silane (3-glycidoxypropyl dimethylethoxysilane, GPDMS, Gelest) surface modification was used in this work [39]. In brief, the GGP-GMR sensor was first treated with oxygen plasma to enrich the TiO2 surface with hydroxyl groups. Subsequently, 1% GPDMS in toluene was dispensed on the sensor surface. Once the biosensor was incubated for 40 min at room temperature, the GPDMS formed covalent bonds with the hydroxyl groups. The GGP-GMR sensor was then rinsed with acetone, IPA, and DI water and blow dried with N_2_ gas. After silanization was performed, the GGP-GMR sensor chip was bonded with the microfluidic channels as described previously.

Albumin and creatinine, commonly used biomarkers for screening microalbuminuria [40,41], exhibit diagnostic efficacy when the urine albumin-to-creatinine ratio exceeds 30 mg/g. These markers were specifically chosen as test models to demonstrate the multiplexing capability of the two-channel microfluidic chip (Figure 1k). A solution comprising 100 μg/mL antialbumin antibodies (CSB-PA00060E1Rb, Cusabio) and anticreatinine antibodies (CRN12-A, Genemed Synthesis) in phosphate-buffered saline (PBS) was injected into each channel. The injected antibody solution only covered one of the GGP-GMR sensors within each channel; these sensors were measurement sensors, and the other sensors without antibodies were reference sensors. After being incubated for 12 h, the antibody solutions were aspirated, and the channels were rinsed using PBS containing 0.05% Tween (PBS-T) thrice to remove unbound antibodies. The channels were then blocked for 1 h at room temperature in a solution comprising 1% casein (ab126587, Abcam, Cambridge, UK) in PBS to minimize nonspecific binding and thus enable subsequent antigen binding. The blocking solution was then aspirated, and the channels were rinsed with PBS-T. Finally, PBS was injected into the channels for measurement to represent a 0% concentration, which served as the baseline signal.

Dose–response curves were generated for various concentrations of albumin (CSB-NP000601h, Cusabio, Houston, TX, USA) and creatinine (02101423-CF, MP Biomedicals, Irvine, CA, USA) in PBS containing 0.1% casein. Albumin solutions of five concentrations from 0.8 to 500 μg/mL with a five-fold dilution were used. The creatinine solution concentrations ranged from 1 μg/mL to 10 mg/mL with a 10-fold dilution. The analyte solutions (starting from the lowest concentration) were injected into the microfluidic channels and incubated for 20 min at room temperature. Once the channels had been rinsed with PBS-T, images were recorded every 10 s for 10 min. This procedure was repeated for the other concentrations.

### 2.4. Detection Principle and Smartphone Readout

The GGP-GMR sensor converts spectral information into spatial information on a CCD. In this work, a smartphone was used to measure the transmission intensity distribution (Figure 4c). An LED (KED351RHD, Kyoto Semiconductor) was used as a light source. The light was transverse-magnetically polarized before it was transmitted through a bandpass filter (Alluxa), resulting in a center wavelength of 630.5 nm with a full width at half maximum of 1.0 nm. The narrowband light source was directed at the PDMS microfluidic channels and interacted with the GGP-GMR sensors. The transmitted light was then focused by a lens and recorded by a smartphone (Sony Xperia XZ1). As discussed previously, the light was reflected back at the resonant period on the sensor corresponding to the sample RI, resulting in dark bands (lower intensity) in the smartphone image. An example is shown in Figure 4d; four dark bands corresponding to the 2 × 2 GGP-GMR sensor array embedded in the two microfluidic channels (Ch1 and Ch2) can be observed.

The intensity distribution along a specific row (i.e., the yellow dashed line) was extracted, and the location of the dark band (or the minimum-intensity pixel) was determined through curve fitting using the Gaussian model in OriginPro 2016 (Figure 4e). By monitoring the shift in the dark band, the sample concentration (or RI) was determined.

## 3. Results

### 3.1. Sucrose Measurement

**Figure 5:**
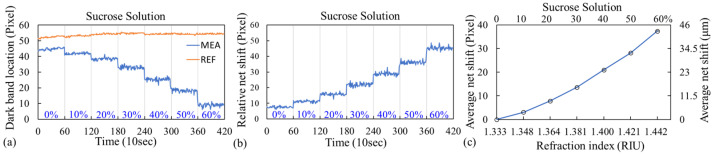
(**a**) Location of the dark bands for different sucrose concentrations for the measurement and reference channels. (**b**) Relative net shift of the dark band with respect to that for the 0% solution. (**c**) Dose–response curve of the sucrose solution.

The image captured by the smartphone contained four dark bands corresponding to a 2 × 2 GGP-GMR sensor array embedded in two microfluidic channels (Figure 4d). When the sample concentration (or RI) changed, the n_eff_ of the structure changed accordingly, resulting in the incident wavelength resonating at a different period, as predicted by Equation (1). Thus, the dark bands also shifted accordingly. Different concentrations of sucrose solutions were used to characterize the bulk sensitivity of the GGP-GMR sensor with a smartphone readout. First, DI water (0%) was injected into both microfluidic channels, and images were taken every 10 s for 10 min. A sucrose solution was then added to one of the microfluidic channels (measurement channel) at concentrations of 10%, up to 60%, in 10% increments. Before sucrose of a certain concentration was injected, the solution was aspirated from the channel, and the channel was rinsed with DI water. The other channel served as a reference channel; only DI water was injected into it. Figure 5a presents the locations of the dark bands for different sucrose concentrations for one of the sensors from both the measurement and reference channels from one of the experimental runs. The *y* axis represents the location of the dark band (or minimum intensity corresponding pixel as illustrated in Figure 4e). Sub-pixel resolution can be obtained through curve fitting. The net shift of the dark band with respect to that for the 0% solution was calculated by subtracting the shift in the dark band of the reference sensor from that of the measurement sensor. The results are presented in Figure 5b. The absolute location of the dark bands in Figure 5a is somehow arbitrary depending on the initial orientation between the sensor chip and the smartphone’s CCD, as well as the chosen location (i.e., yellowed dashed line in Figure 4d) for analysis. Our analysis relies on the relative shift between the reference and the measurement, as shown in Figure 5b. The entire process was repeated for another two runs, and the average net shift in the dark band with respect to that for the 0% solution for each concentration is shown in Figure 5c. The pixel size is 1.15 μm/pixel so the shift in μm is also shown on the right *y* axis in Figure 5c.

The average net shift was 37.89 pixels from the 0% to the 60% solutions; this was equivalent to a total shift of 43.57 μm (at a pixel size of 1.15 μm/pixel). The refractive indices of different concentrations, measured using a commercial refractometer (J47-HA, Rudolph Research Analytical, Hackettstown, NJ, USA), are presented in Figure 5c. The average sensitivity indicates the amount of shift in the dark band for a given change in RI (RI unit; RIU); this was calculated as the slope of the linear-fit line in Figure 5c, which was 393.98 μm/RIU. As recommended by Holstein et al. for experiments with fewer than 10 replicates per concentration level, a pooled standard deviation for all concentrations in the dilution series was used to better represent the population variance [42]. The LOD was then calculated as three times the pooled standard deviation divided by the sensitivity; it was 1.50 × 10^−3^ RIU.

### 3.2. Biomolecule Detection

Albumin and creatinine were used as test models to demonstrate the multiplexing capability of the proposed integrated sensor chip. One channel was used to measure albumin, and the other was used to measure creatinine. Within each channel, the chip had two GGP-GMR sensors; one was a measurement sensor with antibodies, and the other was a reference sensor without antibodies. The four sensors could perform measurements simultaneously (Figure 4d). For albumin or creatinine detection, the measurement was begun by first injecting PBS to stabilize the sensors and acquire a baseline signal (0%); the lowest concentration was then injected and allowed to rest for 20 min. The analytes were then aspirated, PBS-T was used to rinse the channels, and PBS was injected into both channels for the measurement. The image readout and analysis processes were similar to those for the sucrose solution measurement. During the measurement, images of the transmitted intensity distribution were captured every 10 s for 10 min, and the locations of the dark bands were determined as described previously. The same procedure was repeated for other concentrations.

**Figure 6:**
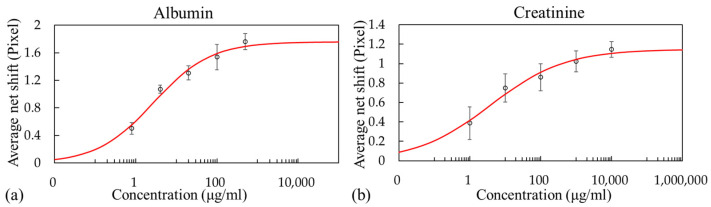
Dose–response curves for the (**a**) albumin and (**b**) creatinine solutions.

The net shift in the dark band caused by the analyte concentration was determined by subtracting the dark band shift of the reference sensor (without antibodies) from that of the measurement sensor (with antibodies) to account for any nonspecific binding and any fluctuations in the measurement system or experimental procedure. The dose–response curves obtained from three experimental runs with three integrated sensor chips are presented in Figure 6. A four-parameter logistic model was developed using OriginPro 2016 to fit the data. The concentration corresponding to a shift of three standard deviations was used to calculate the LOD. The result indicated that for albumin solutions with concentrations of 0.8–500 μg/mL, an LOD of 1.18 μg/mL could be achieved (Figure 6a). For creatinine solutions with concentrations of 1–10 000 μg/mL, the LOD was 20.56 μg/mL (Figure 6b).

The measured shift was less than 2 pixels for albumin and 1 pixel for creatinine; thus, the accuracy of the curve fitting is very important. To mitigate fluctuations in the curve fitting process, for each concentration (i.e., sucrose, albumin, and creatinine), images were captured every 10 s over a period of 10 min (as illustrated in Figure 5a). The average location of the dark band was then employed to represent the dark band location for a specific concentration. This approach helps minimize the potential impact of curve fitting inaccuracies, allowing us to obtain an average net shift with respect to the 0% (blank) for each concentration.

## 4. Discussion

For the sucrose solution, a LOD of 1.50 × 10^−3^ RIU was achieved; this is inferior to those of many RI biosensors reported in the literature. However, an optical biosensor utilizing a photonic crystal waveguide exhibited an LOD of 10^−2^ RIU for bulk solutions and successfully detected human immunoglobulin G with an LOD of 67 nM [43]. The sensing capability demonstrated with the proposed system can be sufficiently effective for the detection of some biomolecules. In addition, preliminary results from the albumin (0.8–500 μg/mL) and creatinine (1–10,000 μg/mL) solutions achieved LODs of 1.18 and 20.56 μg/mL, respectively. It should be noted, however, that the results obtained in this work were from buffer solutions. According to a study with 577 general participants aged more than forty years old from Pakistan, the concentration of albumin in urine was between 2.1 and 8.5 μg/mL [44]. Another study showed the concentration of creatinine in urine was 390–2590 and 280–2170 μg/mL in male and female participants, respectively [45]. While interferences are expected to be encountered when dealing with clinical samples, potentially leading to a reduction in the sensing performance compared to that achieved with buffer solution, it is worth noting that the proposed smartphone biosensor system’s performance can be enhanced by optimizing surface functionalization and assay protocols. This optimization can potentially enable the system to meet the requirements of many clinical applications.

The system’s LOD could be improved through several methods. Many studies have suggested that optimizing the GGP-GMR’s biosensor’s dimensions, including the TiO_2_ thickness and materials, can improve its sensitivity (i.e., increase the dark band shift for a given change of RI) and resonant quality (i.e., narrow the dark band to increase the sensing resolution), thus improving the LOD [46,47]. Additionally, a 2 nm increment in the grating period was employed in this study, owing to the constraints of our e-beam lithography system. Reducing the increment can enhance the detection sensitivity, but it also results in an increase in the sensor size. Further optimization, exploring the number of repeated cycles and increments in the grating period, can be pursued to achieve a higher sensitivity and a narrower dark bandwidth with sufficient resonant efficiency.

According to the simulation results discussed in the previous section, the grating structure with rectangular corners can yield a narrower dark bandwidth to enhance the detection resolution and improve the LOD. The creation of a rectangular grating profile can be achieved through e-beam lithography and reactive ion etching on glass or other low RI substrates. Following the deposition of another higher RI waveguide guiding layer through atomic layer deposition, we anticipate the formation of a rectangular corner grating structure, akin to Figure 3b. This configuration is expected to result in a narrower dark bandwidth, as depicted in Figure 3f, thereby enhancing the sensor performance.

Based on the simulation results from the sinusoidal grating profile shown in Figure 3a, the resonant period shifted from 398.33 to 391.11 nm (Figure 3c), corresponding to sucrose concentrations ranging from 0% to 60%. In addition, at 0%, we could calculate the FWHM as 2.84 nm in terms of the period (396.89 to 399.73 nm, Figure 3c). In this work, the increment was 2 nm, and each period was repeated 100 times. Under the assumption that the CCD is in direct contact with the GGP-GMR sensor, the period shift directly corresponds to the dark band shift in the CCD, resulting in a sensitivity of 1309.72 μm/RIU and a dark bandwidth of 56.58 μm. In contrast, the sensitivity obtained from the experimental results in this study was approximately 393.98 μm/RIU, and the dark bandwidth (as illustrated in Figure 4d,e) at 0% was measured as 22 pixels (equivalent to 25.3 μm). It is evident that the magnification factor introduced by the aspherical and smartphone lens system diminishes the detection sensitivity. However, as long as the dark band remains within the field of view, increasing the magnification factor can enhance the detection sensitivity.

A simple GPDMS surface functionalization was used for antibody immobilization to demonstrate biomolecule detection. However, this resulted in the antibodies having random orientations, reducing the subsequent antigen binding efficiency [48]. Other surface functionalization techniques could be used to achieve higher antibody density or a more favorable orientation, increasing the antigen binding efficiency and further improving the assay detection limit. Furthermore, achieving uniform surface coverage in surface functionalization and antibody immobilization is crucial for detecting low-concentration samples. Further investigation in this regard can be pursued to enhance the overall sensing performance.

As mentioned earlier, the utilization of the average location of the dark band aimed to mitigate the potential impact of curve fitting inaccuracies. Nonetheless, there is still room for improvement. Achieving more accurate curve fitting necessitates a higher quality of the dark band. This entails a preference for a narrower and darker dark band. Such improvements can be realized through device optimization, considering factors such as GGP-GMR dimensions, material selection, or employing a more rectangular grating profile as discussed in the previous section.

Additionally, the optimization of optical components, such as a better collimated light source or a narrower bandwidth of the incident light, can contribute to enhancing the quality of the dark band. The LED used in this work had an average beam angle of 10.45°; if a more collimated light source could be used, a higher resonance quality could be achieved, resulting in a narrower dark band that would enable the resolution of more subtle concentration variations, improving the detection resolution. In addition, a bandpass filter with an FWHM of 1.0 nm was utilized. If a bandpass filter with a narrower FWHM is employed, it could yield a narrower dark bandwidth, thereby enhancing detection resolution.

## 5. Conclusions

In this work, an integrated sensor chip comprising a 2 × 2 GGP-GMR sensor array embedded within two pairs of microfluidic channels was demonstrated for the first time. The sensor can simultaneously detect albumin and creatinine by using a smartphone to directly capture an output image for subsequent analysis. The sensing system achieved a LOD of 1.50 × 10^−3^ RIU for a bulk solution. In addition, the integrated sensor chip and smartphone detection system achieved the simultaneous detection of albumin and creatinine. The LODs were 1.18 and 20.56 μg/mL for albumin and creatinine in buffer solution, respectively.

Once the GGP-GMR sensor array is fabricated, it necessitates a 40 min surface functionalization step, followed by 12 h for antibody immobilization and an additional 1 h blocking process to prepare it for analyte detection. Subsequently, 25 min is required for measurement, including a 20 min analyte incubation period and a 5 min image recording step. The time needed for both sensor preparation and analyte measurement can be further reduced through the optimization of the assay protocol.

The overall detection performance could be further improved by optimizing the following aspects: the GGP-GMR sensor design and fabrication processes, optical setup, antibody immobilization strategy, and assay protocol. The GGP-GMR sensor with smartphone readout represents a new paradigm for label-free biosensor systems and could be useful for many point-of-care applications.

## Data Availability

Data available on reasonable request from the corresponding author.

## References

[1] Kwon L., Long K.D., Wan Y., Yu H., Cunningham B.T. (2016). Medical diagnostics with mobile devices: Comparison of intrinsic and extrinsic sensing. Biotechnol. Adv..

[2] Zhao W.H., Tian S.L., Huang L., Liu K., Dong L.J., Guo J.H. (2020). A smartphone-based biomedical sensory system. Analyst.

[3] Dong S.Y., Guo K.K., Nanda P., Shiradkar R., Zheng G.A. (2014). FPscope: A field-portable high-resolution microscope using a cellphone lens. Biomed. Opt. Express.

[4] Switz N.A., D’Ambrosio M.V., Fletcher D.A. (2014). Low-Cost Mobile Phone Microscopy with a Reversed Mobile Phone Camera Lens. PLoS ONE.

[5] Wang L.J., Chang Y.C., Sun R.R., Li L. (2017). A multichannel smartphone optical biosensor for high-throughput point-of-care diagnostics. Biosens. Bioelectron..

[6] Yu H., Tan Y., Cunningham B.T. (2014). Smartphone Fluorescence Spectroscopy. Anal. Chem..

[7] Zhang D.M., Liu Q.J. (2016). Biosensors and bioelectronics on smartphone for portable biochemical detection. Biosens. Bioelectron..

[8] Zhao H., Liu F., Xie W., Zhou T.C., OuYang J., Jin L., Li H., Zhao C.Y., Zhang L., Wei J. (2021). Ultrasensitive supersandwich-type electrochemical sensor for SARS-CoV-2 from the infected COVID-19 patients using a smartphone. Sens. Actuators B Chem..

[9] Huang L.P., Xiao W., Xu T., Chen H., Jin Z.Y., Zhang Z.G., Song Q.F., Tang Y. (2021). Miniaturized Paper-Based Smartphone Biosensor for Differential Diagnosis of Wild-type Pseudorabies Virus Infection versus Vaccination Immunization. Sens. Actuators B Chem..

[10] Choi C.K., Shaban S.M., Moon B.S., Pyun D., Kim D.H. (2021). Smartphone-assisted point-of-care colorimetric biosensor for the detection of urea via pH-mediated AgNPs growth. Anal. Chim. Acta.

[11] Chen P.C., Chen K.H., Lin C.Y., Yeh Y.C. (2021). Rapidly and simultaneously quantifying multiple biomarkers of L-tyrosine hydroxylase deficiency by using paper microfluidic devices and smartphone-based analysis system. Sens. Actuators B Chem..

[12] Hatiboruah D., Das T., Chamuah N., Rabha D., Talukdar B., Bora U., Ahamad K.U., Nath P. (2020). Estimation of trace-mercury concentration in water using a smartphone. Measurement.

[13] Lai Y.F., Li M.Y., Liao X.F., Zou L. (2021). Smartphone-Assisted Colorimetric Detection of Glutathione and Glutathione Reductase Activity in Human Serum and Mouse Liver Using Hemin/G-Quadruplex DNAzyme. Molecules.

[14] Zhang M.R., Zhang Y., Yang C.K., Ma C.Y., Tang J.G. (2021). A smartphone-assisted portable biosensor using laccase-mineral hybrid microflowers for colorimetric determination of epinephrine. Talanta.

[15] Mudanyali O., Dimitrov S., Sikora U., Padmanabhan S., Navruz I., Ozcan A. (2012). Integrated rapid-diagnostic-test reader platform on a cellphone. Lab Chip.

[16] Zhang J.L., Khan I., Zhang Q.W., Liu X.H., Dostalek J., Liedberg B., Wang Y. (2018). Lipopolysaccharides detection on a grating-coupled surface plasmon resonance smartphone biosensor. Biosens. Bioelectron..

[17] Bremer K., Roth B. (2015). Fibre optic surface plasmon resonance sensor system designed for smartphones. Opt. Express.

[18] Walter J.G., Alwis L.S.M., Roth B., Bremer K. (2020). All-Optical Planar Polymer Waveguide-Based Biosensor Chip Designed for Smartphone-Assisted Detection of Vitamin D. Sensors.

[19] Fan Z.Y., Geng Z.X., Fang W.H., Lv X.Q., Su Y., Wang S.C., Chen H.D. (2020). Smartphone Biosensor System with Multi-Testing Unit Based on Localized Surface Plasmon Resonance Integrated with Microfluidics Chip. Sensors.

[20] Dutta S., Saikia K., Nath P. (2016). Smartphone based LSPR sensing platform for bio-conjugation detection and quantification. RSC Adv..

[21] Huang X.W., Xu D.D., Chen J., Liu J.X., Li Y.B., Song J., Ma X., Guo J.H. (2018). Smartphone-based analytical biosensors. Analyst.

[22] Xu D.D., Huang X.W., Guo J.H., Ma X. (2018). Automatic smartphone-based microfluidic biosensor system at the point of care. Biosens. Bioelectron..

[23] Hou L.L., Duan C.Y., Ding P.P. (2019). Design and Applications of Ratiometric Electrochemical Biosensors. Int. J. Electrochem. Sci..

[24] Preechaburana P., Gonzalez M.C., Suska A., Filippini D. (2012). Surface Plasmon Resonance Chemical Sensing on Cell Phones. Angew. Chem. Int. Edit..

[25] Liu Y., Liu Q., Chen S.M., Cheng F., Wang H.Q., Peng W. (2015). Surface Plasmon Resonance Biosensor Based on Smart Phone Platforms. Sci. Rep..

[26] Roche P.J.R., Filion-Cote S., Cheung M.C.K., Chodavarapu V.P., Kirk A.G. (2011). A Camera Phone Localised Surface Plasmon Biosensing Platform towards Low-Cost Label-Free Diagnostic Testing. J. Sens..

[27] Cetin A.E., Coskun A.F., Galarreta B.C., Huang M., Herman D., Ozcan A., Altug H. (2014). Handheld high-throughput plasmonic biosensor using computational on-chip imaging. Light Sci. Appl..

[28] Gallegos D., Long K.D., Yu H., Clark P.P., Lin Y., George S., Nath P., Cunningham B.T. (2013). Label-free biodetection using a smartphone. Lab. A Chip.

[29] Giavazzi F., Salina M., Ceccarello E., Ilacqua A., Damin F., Sola L., Chiari M., Chini B., Cerbino R., Bellini T. (2014). A fast and simple label-free immunoassay based on a smartphone. Biosens. Bioelectron..

[30] Lin H.A., Huang C.S. (2016). Linear variable filter based on a gradient grating period guided-mode resonance filter. IEEE Photonics Technol. Lett..

[31] Hsiung C.T., Huang C.S. (2019). Refractive Index Sensor Based on a Gradient Grating Period Guided-Mode Resonance. IEEE Photonics Technol. Lett..

[32] Lin H.C., Wang Y.C., Yang J.M., Huang C.S., Kuo S.H., Li B.R. (2021). Gradient Grating Period Guided-Mode Resonance for Potential Biosensing Applications. IEEE Sens. J..

[33] Guner H., Ozgur E., Kokturk G., Celik M., Esen E., Topal A.E., Ayas S., Uludag Y., Elbuken C., Dana A. (2017). A smartphone based surface plasmon resonance imaging (SPRi) platform for on-site biodetection. Sens. Actuators B Chem..

[34] Wang S.S., Magnusson R. (1993). Theory and applications of guided-mode resonance filters. Appl. Opt..

[35] Wang S.S., Magnusson R. (1994). Design of wave-guide-grating filters with symmetrical line-shapes and low side-band. Opt. Lett..

[36] Sharon A., Rosenblatt D., Friesem A.A. (1997). Resonant grating waveguide structures for visible and near-infrared radiation. J. Opt. Soc. Am. A Opt. Image Sci. Vis..

[37] Magnusson R., Ding Y., Lee K.J., Shin D., Priambodo P.S., Young P.P., Maldonado T.A., Eldada L.A. (2003). Photonic devices enabled by waveguide-mode resonance effects in periodically modulated films. Nano-And Micro-Optics for Information Systems, Proceedings of the Society of Photo-Optical Instrumentation Engineers (Spie).

[38] Xiong L.C., Chen P., Zhou Q.S. (2014). Adhesion promotion between PDMS and glass by oxygen plasma pre-treatment. J. Adhes. Sci. Technol..

[39] Dorvel B., Reddy B., Block I., Mathias P., Clare S.E., Cunningham B., Bergstrom D.E., Bashir R. (2010). Vapor-Phase Deposition of Monofunctional Alkoxysilanes for Sub-Nanometer-Level Biointerfacing on Silicon Oxide Surfaces. Adv. Funct. Mater..

[40] Mattix H.J., Hsu C.Y., Shaykevich S., Curhan G. (2002). Use of the albumin/creatinine ratio to detect microalbuminuria: Implications of sex and race. J. Am. Soc. Nephrol..

[41] Price C.P., Newall R.G., Boyd J.C. (2005). Use of protein: Creatinine ratio measurements on random urine samples for prediction of significant proteinuria: A systematic review. Clin. Chem..

[42] Holstein C.A., Griffin M., Hong J., Sampson P.D. (2015). Statistical Method for Determining and Comparing Limits of Detection of Bioassays. Anal. Chem..

[43] Pal S., Guillermain E., Sriram R., Miller B.L., Fauchet P.M. (2011). Silicon photonic crystal nanocavity-coupled waveguides for error-corrected optical biosensing. Biosens. Bioelectron..

[44] Jafar T.H., Chaturvedi N., Hatcher J., Levey A.S. (2007). Use of albumin creatinine ratio and urine albumin concentration as a screening test for albuminuria in an Indo-Asian population. Nephrol. Dial. Transplant..

[45] Songjaroen T., Maturos T., Sappat A., Tuantranont A., Laiwattanapaisal W. (2009). Portable microfluidic system for determination of urinary creatinine. Anal. Chim. Acta.

[46] Ju J., Han Y.-A., Kim S.-M. (2013). Design Optimization of Structural Parameters for Highly Sensitive Photonic Crystal Label-Free Biosensors. Sensors.

[47] Wan Y.H., Krueger N.A., Ocier C.R., Su P., Braun P.V., Cunningham B.T. (2017). Resonant Mode Engineering of Photonic Crystal Sensors Clad with Ultralow Refractive Index Porous Silicon Dioxide. Adv. Opt. Mater..

[48] Welch N.G., Scoble J.A., Muir B.W., Pigram P.J. (2017). Orientation and characterization of immobilized antibodies for improved immunoassays. Biointerphases.

